# EEG Waveform Analysis of P300 ERP with Applications to Brain Computer Interfaces

**DOI:** 10.3390/brainsci8110199

**Published:** 2018-11-16

**Authors:** Rodrigo Ramele, Ana Julia Villar, Juan Miguel Santos

**Affiliations:** Computer Engineering Department, Instituto Tecnológico de Buenos Aires (ITBA), Buenos Aires 1441, Argentina; jvillar@itba.edu.ar (A.J.V.); jsantos@itba.edu.ar (J.M.S.)

**Keywords:** electroencephalography, brain-computer interfaces, waveform, p300, SIFT, PE, MP, SHCC

## Abstract

The Electroencephalography (EEG) is not just a mere clinical tool anymore. It has become the de-facto mobile, portable, non-invasive brain imaging sensor to harness brain information in real time. It is now being used to translate or decode brain signals, to diagnose diseases or to implement Brain Computer Interface (BCI) devices. The automatic decoding is mainly implemented by using quantitative algorithms to detect the cloaked information buried in the signal. However, clinical EEG is based intensively on waveforms and the structure of signal plots. Hence, the purpose of this work is to establish a bridge to fill this gap by reviewing and describing the procedures that have been used to detect patterns in the electroencephalographic waveforms, benchmarking them on a controlled pseudo-real dataset of a P300-Based BCI Speller and verifying their performance on a public dataset of a BCI Competition.

## 1. Introduction

Current society is demanding technology to provide the means to realize the utopia of social inclusion for people with disabilities [1]. Additionally, as societies are aging [2] the incidence of neuromuscular atrophies, strokes and other invalidating diseases is increasing. Concurrently, the digital revolution and the pervasiveness of digital gadgets have modified the way people interact with the environment through these devices [3]. All this human computer interaction is based on muscular movement [4], but these trends are pushing this boundary beyond the confines of the body and beyond the limitation of human motion. A new form of human machine communication which directly connects the Central Nervous System (CNS) to a machine or computer device is currently being developed: Brain Machine Interfaces (BMI), Brain Computer Interfaces (BCI) or Brain-Neural Computer Interfaces (BNCI).

At the center of all this hype, we can find a hundredth year old technology, rock-solid as a diagnosis tool, which greatly benefited from the shrinkage of sensors, the increase in computer power and the widespread development of wireless protocols and advanced electronics: the Electroencephalogram (EEG) [5].

EEG sensors are wearable [6] non-invasive, portable and mobile [7], with excellent temporal resolution, and acceptable spatial resolution [8]. This humble diagnosis device is been transformed into currently the best approach to detect, out-of-the lab in an ambulatory context, information from the Central Nervous System and to use that information to volitionally drive cars, steer drones, write emails, control wheelchairs or to assess alcohol consumption [9,10,11,12].

The clinical and historical tactic to analyze EEG signals is based on detecting visual patterns out of the EEG trace or polygraph [8]: multichannel signals are extracted and continuously plotted over a piece of paper. Electroencephalographers or Electroencephalography technician decode and detect patterns along the signals by visually inspecting them [5]. Nowadays clinical EEG still remains a visually interpreted test [8].

The need of quantitative procedures to automate the decoding of EEG signals has been materialized in BCI where around 71.2% is based on noninvasive EEG [4]. However, methods of decoding signals based on the detection of waveforms has been scarce. Hence, the traditional and knowledgeable approach has been neglected particularly in BCI Research. We aim to help fix this gap by providing a review of the methods which emphasize the waveform, the shape of the EEG signal and which can decode them in a supervised and semi-automated procedure.

The aim of this study is threefold: first to review current literature of EEG processing techniques which are based on analysis of the waveform. The second is to evaluate and study these methods by analyzing its classification performance against a pseudo-real dataset. And third, to verify their applicability to a real and public dataset.

This article unfolds as follows: Section 2 provides a brief introduction to EEG and the particularities of the EEG waveform characterization. Section 3.1 explains the waveform-based algorithms that are analyzed. In Section 3.6 the experimentation procedure is explained. Results are presented in Section 4 and finally Discussion and Conclusions are expounded in the final sections.

## 2. Electroencephalography

The Electroencephalography consists on the measurement of small variations of electrical voltage over the scalp. It is one of the most widespread used methods to capture brain signals and was initially developed by Hans Berger in 1924 and has been extensively used for decades to diagnose neural diseases and other medical conditions.

The first characterization that Dr. Berger detected was the Visual Cortical Alpha Wave, the *Berger Rythm* [13]. He understood that the amplitude and shape of this rhythm was coherently associated to a cognitive action (eyes closing). We should ask ourselves if the research advancement that came after that discovery would have happened if it weren’t so evident that the shape alteration was due to a very simple and verifiable cognitive process.

**Figure 1:**
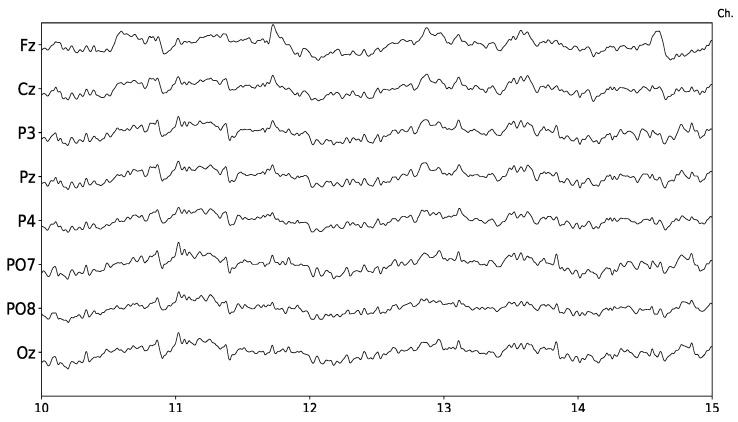
Sample EEG signal obtained from g.Tec g.Nautilus. Time axis is in seconds and five seconds are displayed. The eight channels provided by this device are shown.

The EEG signal is a highly complex multi-channel time-series. It can be modeled as a linear stochastic process with great similarities to noise [14]. It is measured in microvolts, and those slightly variations are contaminated with heavy endogenous artifacts and exogenous spurious signals. Figure 1 shows 5 s of a sample 8-channel EEG signal.

The device that captures these small variations in potential differences over the scalp is called the Electroencephalograph. Electrodes are located in predetermined positions over the head, usually embedded in saline solutions to facilitate the electrophysiological interface and are connected to a differential amplifier with a high gain which allows the measurement of tiny signals. Although initially analog devices were developed and used, nowadays digital versions connected directly to a computer are pervasive. A detailed explanation on the particularities and modeling of EEG can be obtained from [15], and a description of its electrophysiological aspects from [16].

Overall, EEG signals can be described by their phase, amplitude, frequency and *waveform*. The following elements regularly characterize EEG signals:

- Artifacts: These are signal sources which are not generated from the CNS, but can be detected from the EEG signal. They are called endogeneous or physiological when they are generated from a biological source like face muscles, ocular movements, etc., and exogeneous or non-physiological when they have an external electromagnetic source like line induced currents or electromagnetic noise [17].
- Non-Stationarity: the statistical parameters that describe the EEG as a random process are not conserved through time, i.e., its mean and variance, and any other higher-order moments are not time-invariant [13].
- DC drift and trending: in EEG jargon, which is derived from concepts of electrical amplifiers theory, Direct Current (DC) refers to very low frequency components of the EEG signal which varies around a common center, usually the zero value. DC drift means that this center value drifts in time. Although sometimes considered as a nuisance that needs to get rid of, it is known that very important cognitive phenomena like Slow Cortical Potentials or Slow Activity Transients in infants do affect the drift and can be used to understand some particular brain functioning [5].
- Basal EEG activity: the EEG is the compound summation of myriads of electrical sources from the CNS. These sources generate a baseline EEG which shows continuous activity with a small or null relation with any concurrent cognitive activity or task.
- Inter-subject and intra-subject variability: EEG can be affected by the person’s behavior like sleep hygiene, caffeine intake, smoking habit or alcohol intake previously to the signal measuring procedure [18].

Regarding how the EEG activity can be related to an external stimulus that is affecting the subject, it can be considered as

- Spontaneous: generally treated as noise or basal EEG.
- Evoked: activity that can be detected synchronously after some specific amount of time after the onset of the stimulus. This is usually referred as time-locked. In contrast to the previous one, it is often called Induced activity.

Additionally, according to the existence of a repeated rhythm, the EEG activity can be understood as

- Rhythmic: EEG activity consisting in waves of approximately constant frequency. It is often abbreviated RA (regular rythmic activity). They are loosely classified by their frequencies, and their naming convention was derived from the original naming used by Hans Berger himself, and after Alpha Waves (10 Hz), it came Delta (4 Hz), Theta (4–7 Hz), Sigma (12–16 Hz), Beta (12–30 Hz) and Gamma (30–100 Hz).
- Arrhythmic: EEG activity in which no stable rhythms are present.
- Dysrhythmic: Rhythms and/or patterns of EEG activity that characteristically appear in patient groups and rarely seen in healthy subjects.

The number of electrodes and their positions over the scalp determines a **Spatial Structure**: signal elements can be generalized, focal or lateralized, depending on in which channel (i.e., electrode) they are found.

### EEG Waveform Characterization

The shape of the signal, the waveform, can be defined as the graphed line that represents the signal’s amplitude plotted against time. It can also be called EEG biomarker, EEG pattern, signal shape, signal form and a morphological signal [13].

The signal context is crucial for waveform characterization, both in a spatial and in a temporal domain [13]. Depending on the context, some specific waveform can be considered as noise while in other cases is precisely the element which has a cognitive functional implication.

A waveform can have a characteristic shape, a rising or falling phase, a pronounced plateau or it may be composed of ripples and wiggles. In order to describe them, they are characterized by its amplitude, the arch, whether they have (non)sinusoidal shape, by the presence of an oscillation or imitating a sawtooth (e.g., Motor Cortical Beta Oscillations). The characterization by their sharpness is also common, particularly in Epilepsy, and they can also be identified by their resemblance to spikes (e.g., Spike-wave discharge).

Depictions may include subjective definitions of sharper, arch comb or wicket shape, rectangular, containing a decay phase or voltage rise, peaks and troughs, short term voltage change around each extrema in the raw trace. Derived ratios and indexes can be used as well, like peak and trough sharpness ratio, symmetry between rise and decay phase and slope ratio (steepness of the rise period to that of the adjacent decay period). For instance, wording like “Central trough is sharper and more negative that the adjacent troughs” [19] are common in the literature.

Other regular characterizations which are based on the waveform shape may encompass:

- Attenuation: Also called suppression or depression. Reduction of amplitude of EEG activity resulting from decreased voltage. When activity is attenuated by stimulation, it is said to have been “blocked” or to show “blocking”.
- Hypersynchrony: Seen as an increase in voltage and regularity of rhythmic activity, or within the alpha, beta, or theta range. The term suggest an increase in the number of neural elements contributing to the rhythm, or in the synchronization of different neurons with the same discharge pattern [20].
- Paroxysmal: Activity that emerges from background with a rapid onset, reaching frequently high voltage and ending with an abrupt return to lower voltage activity.
- Monomorphic: Activity appearing to be composed of one dominant waveform pattern.
- Polymorphic: Activity composed of multiple frequencies that combine to form a complex waveform.
- Transient/Component: An isolated wave or pattern that is distinctly different from background activity.

The traditional clinical approach to study electroencephalographic signals consists in analyzing the paper strip that is generated by the plot of the signal obtained from the device. Expert technician and physicians analyze visually the plots looking for specific patterns that may give a hint of the underlying cognitive process or pathology. Atlases and guidelines were created in order to help in the recognition of these complex patterns. Video-electroencephalography scalp recordings are routinely used as a diagnostic tools [21] . The clinical EEG research has also focused on temporal waveforms, and a whole branch of electrophenomenology has arisen around EEG *graphoelements* [5].

Sleep Research has been studied in this way by performing Polysomnographic recordings (PSG) [22,23]. The different sleep stages are evaluated by visually marking waveforms or graphoelements in long-running electroencephalographic recordings, looking for patterns based on standardized guidelines [24]. Visual characterization includes the identification or classification of certain waveform components based on a subjective characterization (e.g., positive or negative peak polarity) or the location within the strip. It is regular to establish an amplitude difference between different waveforms from which a relation between them is reckoned and a structured index is created (e.g., sleep K-Complex is well characterized based on rates between positive vs negative amplitude) [25]. Other relevant EEG patterns for sleep stage scoring are alpha, theta, and delta waves, sleep spindles, polysplindles, vertex sharp waves (VSW), and sawtooth waves (REM Sleep).

Moreover, EEG data acquisition is a key procedure during the assessment of patients with focal Epilepsy for potential seizure surgery, where the source of the seizure activity must be reliably identified. The onset of the Epileptic Seizure is defined as the first electrical change seen in the EEG rhythm which can be visually identified from the context and it is verified against any clinical sign indicating seizure onset. The Interictal Epileptiform Discharges (IEDs) are visually identified from the paper strip, and they are also named according to their shape: spike, spike and wave or sharp-wave discharges [26].

Waveform characterization is the method in which analysis has been performed for Event Related Potentials (ERP). These are transient signal elements that may arise as a brain response to an external visual, tactile or auditory stimulus. ERPs are regularly used to assess auditory response in infants. They are extensively used and studied in Cognitive Neuroscience [27]. ERPs are identified by their components which are recognizable signal shapes assigned to the observed waveform, that can be linked to some cognitive or measurable psychological process. One of the most studied ERP is the P300, discovered in 1965 by Sutton, Braren, Zubin and John. This component is a positive deflection of a subject’s EEG signal that arises when an unexpected and infrequent stimulus appears [1]. The P300 is widely utilized in BCI because it can be harnessed to implement a Speller application. Hence, P300 ERPs are a target phenomena to study by automatic waveform recognition methods.

Table 1 summarizes a list of depictions used to describe waveforms in the surveyed literature. Epilepsy has been described by the nature of oscillatory characterization of their waves, like ripples and wiggles, imitating sawtooths or by their geometric shape. For ERPs on the other hand, more elaborate indexes has been provided, establishing relations between amplitudes of signal components. Finally, Sleep studies and ICU research are areas where the most complex indexes has been derived, particularly the coupling of signal properties like phase, amplitude and frequency.

## 3. Materials and Methods

The exploration of methods based on waveforms is conducted by following the PRISMA [39] guidelines. Search is performed on Google Scholar, Semantic Web and IEEE Xplore search engines by the terms “Waveforms” OR “Shape” OR “Morphology” OR “Visual inspection” + “EEG”.

The following criteria is proposed to identify methods which are based on the signal’s waveform:

1. The analysis considers the shape of the plot of the signal.
2. The pattern can be identified and verified by visual inspection.
3. The pattern matching is performed in time-domain.
4. The method encompass a feature extraction procedure.
5. The feature extraction procedure allows to create a template dictionary.

As described in [40] the Pattern Matching problem in Signal processing is finding a signal given the region that best describes the structure of the prototype signal template. On the other hand, a *feature* is a meaningful quantification, usually a multidimensional vector, that synthesize the information of a given signal or signal segment [41].

### 3.1. EEG Waveform Analysis Algorithms

Shape or waveform analysis methods are considered as nonparametric methods. They explore signal’s time-domain metrics or even derive more complex indexes or features from it [42].

One of the earliest approach to automatically process EEG data is the Peak Picking method. Although of limited usability, peak picking has been used to determine latency of transient events in EEG [43,44]. Straightforward in its implementation, it consists in assigning a component to a simple waveform element based on the expected location of its more prominent deflection [31]. Of regular use in ERP Research, the name of many of the EEG features reference directly a peak within the component, e.g., P300 or P3a P3b or N100. This leads to a natural way to classify them visually by selecting appropriate peaks and matching their positions and amplitudes in an orderly manner. The letter provides the polarity (Positive or Negative) and the numbering shows the time referencing the stimulus onset, or the ordinal position of each peak (first, second, etc).

A related method is used in [45] where the area under the curve of the EEG is sumarized to derive a feature. This was even used in the seminal work of Farwell and Donchin on the P300 Speller [41,46]. Additionally, a logarithmic graph of the peak-to-peak amplitude which is called amplitude integrated EEG (aEEG) [38] is used nowadays in Neonatal Intensive Care Units.

Other works on EEG explored the idea to extend human capacities analyzing EEG waveforms. In [47] a feature derived from the amplitude and frequency of its signal and its derivative in time-domain is used. Moreover, Yamaguchi et al. [48] explored the use of Mathematical Morphology, where the time-domain structure of contractions and dilations were studied. Finally the proposals of Burch, Fujimori, Uchida and the Period Amplitude Analysis (PAA) [49] algorithm are few of the earliest depictions where the idea of capturing the shape of the signal were established.

According to the defined criteria, the algorithms that will be evaluated are as follows:

- Matching Pursuit
- Permutation Entropy
- Slope Horizontal Chain Code
- Scale Invariant Feature Transform

All these methods provide a feature *f* that can be used as a template. The notation $f={\left\{f_{i}\right\}}_{1}^{n}$ or $f={\left\{f_{i}\right\}}_{i\in J}$ is used to describe the concatenation of scalar values to form a multidimensional feature vector $f=\{f_{1},f_{2},\;\ldots ,\;f_{n}\}$. These algorithms are all based on metrics that are extracted from the shape of the single channel digital EEG signal x(n), with *n* varying from 1 to the length *N* of the EEG segment in sample points. These features are used to create dictionaries or template databases. Finally, these templates provide the basis for the pattern matching algorithm and offline classification. Algorithms were implemented on MATLAB 2014a (Mathworks Inc., Natick, MA, USA). To maintain reproducibility, the dataset described in Section 3.6.1 and the source code has been made available in the online repository of the Code Ocean platform under the name *EEGWave*.

### 3.2. Matching Pursuit—MP 1 and MP 2

*Pursuit* algorithms refer, in their many variants, as blind source separation [50] techniques that assume the EEG signal as a linear combination of different sparse sources extracted from a template’s dictionaries. Matching Pursuit *MP* [51], the most representative of these algorithms, is a greedy variant that decomposes a signal into a linear combination of waveforms, called atoms, that are well localized in time and frequency [52]. Given a signal, this optimization technique, tries to find the indexes of *m* atoms and their weights (contributions) that minimize,

(1) $$\varepsilon =∥x\left(n\right)-\sum_{i=1}^{m}w_{i}g_{i}\left(n\right)∥$$

which is the error between the signal and its approximation constructed by the weighted $w_{i}$ atoms $g_{i}$, and calculating the euclidean norm ${∥\cdot ∥}_{2}$. The algorithm goes by first setting the approximating signal ${\tilde{x}}_{0}$ as the original signal itself,

(2) $${\tilde{x}}_{0}\left(n\right)=x\left(n\right)$$

and setting the iterative counter *k* as 1. Hence, it searches recurrently the best template out of the dictionary that matches current approximation.

(3) $$g_{k}=\arg\max_{g_{i}}\left|\sum_{n=1}^{N}{\tilde{x}}_{k-1}\left(n\right)\;g_{i}\left(n\right)\right|$$

where $g_{i}$ are all the available scaled, translated and modulated atoms from the dictionary. The operation $\left|\cdot \right|$ corresponds to the absolute value of the inner product. This step determines the atom selection process, and their contribution is calculated based on

(4) $$w_{k}=\frac{\sum_{n=1}^{N}{\tilde{x}}_{k-1}\left(n\right)\;g_{k}\left(n\right)}{{∥g_{k}∥}^{2}}$$

with *k* representing the index of the selected atom $g_{k}$ and ${∥\cdot ∥}_{2}$ its euclidean norm. Finally the contribution of each atom is subtracted from the next approximation [32,51,53]

(5) $${\tilde{x}}_{k}\left(n\right)={\tilde{x}}_{k-1}\left(n\right)-w_{k}g_{k}\left(n\right)$$

The stopping criteria can be established based on a limiting threshold on Equation (1) or based on a predetermined number of steps and selected atoms. Two variants of this algorithm are evaluated. In *MP 1* the dictionary is constructed with the normalized templates directly extracted from the real signal segments which is a straightforward implementation of the pattern matching technique. In *MP 2* the coefficients of Daubechies least-asymetric wavelet with 2 vanishing moments atoms are used to construct the dictionary [54]. For the first version, the matching against the template is evaluated according to Equation (1) directly, whereas for the latter each feature is crafted by decomposing the signal in its coefficients and building, an eventually sparse, vector with them:

(6) $$f={\left\{w_{i}\right\}}_{1}^{D}$$

where *D* is the size of the dictionary.

### 3.3. Permutation Entropy—PE

Bond and Pompe Permutation Entropy has been extensively used in EEG processing, with applications on Anesthesia, Sleep Stage evaluation and increasingly for Epilepsy pre-ictal detection [55]. This method generates a code based on the orderly arrangement of sequential samples, and then derives a metric which is based on the number of times each sequence is found along the signal. This numeric value can be calculated as information entropy [56]. Let’s consider a signal on a window of length *W* represented by the sample points

(7) $$\left(x_{1},\;x_{2},\;\ldots ,\;x_{W}\right)$$

and resampled by τ intervals, starting from the sampling point *n*, doing

(8) $$(x_{n},\;x_{n+\tau },\;x_{n+2\tau }\ldots ,\;x_{n+(m-1)\tau }).$$

This sequence is of order *m*, which is the number of sample points used to derive the ordinal element called π. There are m! ways in which this sequence can be orderly arranged, according to the position that each sample point holds within the sequence in a decreasing order relationship [57]. For example if m=3, and the first sample point is the bigger, the second is the smaller and the third one is in the middle, the ordinal element π corresponds to (1, 3, 2). Thus, along the signal window there can be at most *k* different ordinal (and overlapping) elements $\pi_{s}$

(9) $$\left(\pi_{1},\;\pi_{2},\;\ldots ,\;\pi_{k}\right)$$

with k=W−(m−1)τ. The probability density function *pdf* for all the available permutations of order *m* should be $p=(p_{1},\;p_{2},\;\ldots ,\;p_{m!})$ with $\sum_{i=1}^{m!}p_{i}=1$.

Hence, the time series window is mapped to a new set of *k* ordinal elements, and the *pdf* can be calculated by the empirical permutation entropy,

(10) $$p_{i}=\frac{1}{k}\sum_{s=1}^{k}\left[\pi_{s}=\pi_{i}\right]$$

with 1≤i≤m!. The Iverson Bracket $\left[\cdot \right]$ resolves to 1 when their logical proposition argument is true, 0 otherwise. Therefore, for each *i* only those ordinal elements $\pi_{s}$ that were effectively found along the signal are counted to estimate $p_{i}$, and zero elsewhere. The empirical permutation entropy can be calculated from the histogram as,

(11) $$H\left(p\right)=\sum_{i=1}^{m!}p_{i}\;log\frac{1}{p_{i}}.$$

The implemented code was derived from [58], and the model description from [59]. This procedure produces a scalar number for the given signal window of size *W*. To derive a feature, a sliding window procedure must be implemented to cover an entire segment of length *N*. Thus, the length of the feature is N−(W+τ(m−1)).

(12) $$f={\left\{H{\left(p\right)}_{u}\right\}}_{W+\tau m}^{N}.$$

with *u* varying on a sample by sample basis along the signal, starting from the specified index.

### 3.4. Slope Horizontal Chain Code—SHCC

This algorithm [45] proceeds by generating a coding scheme from a sequence of sample points. This encoding is based on the angle between the horizontal line on a 2D-plane and any segment produced by two consecutive sample points, regarding them as coordinates on that plane.

A signal of length *N*, can be represented by a list of ordered-pairs *e*,

(13) $$e=\left[{\left(x,\;y\right)}_{1},\;{\left(x,\;y\right)}_{2},\;\ldots ,\;{\left(x,\;y\right)}_{N}\right]$$

and it can be divided into *G* different blocks. These blocks are obtained by resampling the original signal from the index

(14) G=⌊n+(mΔ)+0.5⌋

with *n* being the original sampling index on 1≤n≤N and ⌊·⌋ being the floor operation, i.e., rounding of the number argument to the closest smaller integer number. On the other hand, Δ can be obtained by

(15) $$\Delta =\left\lceil \frac{N}{G+1}\right\rceil$$

with G<N and using instead ⌈·⌉ as the ceil operation, the rounding to the closest bigger integer number. Lastly, the value *m* can be derived from

(16) $$m=sign\left(\frac{N-1}{\Delta }\right)\left\lfloor \left|\frac{N-1}{\Delta }\right|\right\rfloor .$$

This resampling produces a new sequence of values,

(17) $$e^{\prime }=\left[{\left(x^{\prime },\;y^{\prime }\right)}_{1},\;\ldots ,\;{\left(x^{\prime },\;y^{\prime }\right)}_{s},\;\ldots ,\;{\left(x^{\prime },\;y^{\prime }\right)}_{G}\right].$$

The next step is the normalization of each ordered-pair as vectors $x^{\prime }=\left(x_{1}^{\prime },\;\ldots ,\;x_{G}^{\prime }\right)$ and $y^{\prime }=\left(y_{1}^{\prime },\;\ldots ,\;y_{G}^{\prime }\right)$ according to

(18) $$\hat{x}=\frac{x^{\prime }-\min\left(x^{\prime }\right)1}{\max\left(x^{\prime }\right)-\min\left(x^{\prime }\right)}$$

(19) $$\hat{y}=\frac{y^{\prime }-\min\left(y^{\prime }\right)1}{\max\left(y^{\prime }\right)-\min\left(y^{\prime }\right)}$$

with 1 being the vector of length *G* with all their components equal to 1. Hence, the scalar components ${\hat{x}}_{s}$ of $\hat{x}$, and ${\hat{y}}_{s}$ of $\hat{y}$, with *s* varying between 1 and *G* are effectively normalized to ${\hat{x}}_{s},\;{\hat{y}}_{s}\in \left[0,\;1\right]$.

Finally, the feature is constructed by calculating the point-to-point slope against the horizontal plane,

(20) $$f={\left\{\frac{{\hat{y}}_{s}-{\hat{y}}_{s-1}}{{\hat{x}}_{s}-{\hat{x}}_{s-1}}\right\}}_{2}^{G}$$

### 3.5. Scale Invariant Feature Transform—SIFT

SIFT [60] is a very successful feature extraction technique from Computer Vision. It has a biomimetic inspiration on how the visual cortex analyze images based on orientations [61]. This method has been used in [62] to analyze EEG signals based on their plots on 2D images.

The first step of the algorithm is the plot generation based on single-channel EEG segments x(n). Hence, this signal is normalized by the z-score [63]:

(21) $$\tilde{x}\left(n\right)=\left\lfloor \frac{\delta (x\left(n\right)-\bar{x})}{\sigma_{x}}\right\rceil$$

with δ being the signal magnification factor and $\bar{x}$ and $\sigma_{x}$, the mean and standard deviation of *x* on the signal segment. The width of the image is determined based on the 1-s length size of the segment in sample units. This corresponds to the sampling frequency $F_{s}$ of the EEG signal segment. The width is adjusted by multiplying by the magnification factor δ,

(22) $$w=\delta \;F_{s}$$

whereas the height is calculated based on the peak-to-peak amplitude of the signal within the segment,

(23) $$h=\max_{n}\tilde{x}\left(n\right)-\min_{n}\tilde{x}\left(n\right).$$

Equation (24) determines the vertical position of the image where the signal’s zero value will be located.

(24) $$z=\left\lfloor \frac{h}{2}\right\rfloor -\left\lfloor \frac{\max_{n}\tilde{x}\left(n\right)+\min_{n}\tilde{x}\left(n\right)}{2}\right\rfloor .$$

Finally, a binary, black-and-white image plot is generated based on

(25) $$I\left(z_{1},z_{2}\right)=\left\{\begin{array}{cc} 255 & \mathrm{if}\;z_{1}=\delta \;n;\;z_{2}=\tilde{x}\left(n\right)+z\\ 0 & \mathrm{otherwise} \end{array}\right.$$

where $z_{1}$ and $z_{2}$ are the image coordinates values, 255 represents white and 0 is the background black color of the plot. These points are interpolated using the Bresenham algorithm [62].

Once the plot is generated, its center is used to localize the center of the SIFT patch. This region of the image, where the signal’s most important salient waveform should be located, is divided in a grid of 4×4 block and the bidimensional gradient vectors are calculated on each one of them. Therefore, for each block (i,j) within the patch, a histogram h(i,j,θ) of the gradient orientations, for 8 circular orientations θ, are calculated. This histogram is concatenated for all the 16 blocks and a feature is thus formed:

(26) $$f={\left\{{\left\{{\left\{h(i,\;j,\;\theta )\right\}}_{i\in I}\right\}}_{j\in I}\right\}}_{\theta \in \Theta }$$

with *i* and *j* belonging to I={0, 1, 2, 3} and localizing the 16 blocks within the grid. The angles θ that belong to Θ are the eight possible equidistant values between 0 and 315. This vector is normalized, clamped to 0.2, and re-normalized again. Details of the method can be found on [60,62]. It was implemented using the VLFeat [64] public Computer Vision libraries.

### 3.6. Experimental Protocol

The objective of the following experiments is to assess the performance of the algorithms that aim to recognize the shape of the P300 waveform, obtained after averaging signal segments. This performance is evaluated by processing a pseudo-real dataset with two modalities where subtle alterations on the latency and amplitude of the P300 component are simulated in a controlled environment. The experiments are performed by the offline evaluation of the character identification rate of a Visual P300-Based BCI Speller application.

Farwell and Donchin P300 Speller [46,65] is one the most used BCI paradigms to implement a thought translation device and to send commands to a computer in the form of selected letters, similar to typing on a virtual keyboard. This procedure exploits a cognitive phenomena raised by the oddball paradigm [27]: along the EEG trace of a person which is focusing on a sequence of two different visual flashing stimulus, a particular and distinctive transient component is found each time the expected stimulus flashes. This is cleverly utilized in the P300 Speller, where rows and columns of a 6 × 6 matrix flashes randomly but only the flashing of a column or row where the letter that a user is focusing will trigger concurrently the P300 ERP along the EEG trace.

A problem with the information produced by a P300 Speller is that the subjects that take part on the experiment are within the closed loop of the BCI system and the human is not a static compliant entity that always performs what the experimenter asks for in a precise and consistent way [66]. Therefore, P300 experiments data is often mined with *null-signals*. These are EEG streams which are marked as having the signal component but, because the subject was not particularly focused, or concentrated, the expected signal element is not generated. This lack of certainty may be in detriment of any conducted analysis and can be misleading or difficult to deal with. Previous works have addressed this same issue, particularly when benchmarking different algorithms [31,43,67].

In order to tackle this problem, a pseudo-real dataset based on an EEG stream is generated under two different modalities. A passive modality and an active modality.

#### 3.6.1. EEG Stream Generation

Eight (8) healthy participants are recruited voluntarily and the experiment is conducted anonymously in accordance with the Declaration of Helsinki published by the World Health Organization. No monetary compensation is handed out and they agree and sign a written informed consent. This study is approved by the *Departamento de Investigación y Doctorado, Instituto Tecnológico de Buenos Aires (ITBA)*. The participants are healthy and have normal or corrected-to-normal vision and no history of neurological disorders. These voluntary subjects are aged between 20–40 years old. EEG data is collected in a single recording session. Each subject is seated in a comfortable chair, with her/his vision aligned to a computer screen located one meter in front of her/him. The handling and processing of the data and stimuli is conducted by the OpenVibe platform [68]. Gel-based active electrodes (g.LADYbird, g.Tec, Austria) are used on locations Fz, Cz, Pz, Oz, P3,P4, PO7 and PO8 according to the 10–20 international system. Reference is set to the right ear lobe and ground is preset as the AFz position. Sampling frequency is set to 250 Hz.

The experimental protocol is composed of 35 trials to spell 7 words of 5 letters each. Each trial is composed of 10 intensification sequences of the 6 columns and 6 rows of the Speller Matrix. This yields exactly 120 intensifications of rows and columns per trial. The duration of each intensification as well as the Inter-Stimulus Interval, the pause between stimulations, are set to 0.125 s. This provides a 4 Hz frequency of flashes on the screen. The initial pause and the inter-trial pauses are set to 20 s. The whole experiment lasts for around 1400 s. This produces an EEG stream which contains 4200 marked sections where 3500 of them are labeled as *True* and the remaining 700 as *False*. The extracted EEG signals are band-pass filtered using a 4th order Butterworth digital filter between 0.1 and 10 Hz and a 50 Hz notch filter is applied to remove line AC noise. The EEG trace is finally downsampled to 16 Hz. Segments of 1-s length are extracted according to the markers information and those with variations larger than 70 μV are identified as artifacts and eliminated.

Four out of the eight participants are instructed to passively watch the flashing screen while not focusing on any particular letter. They do not receive any extra information on the screen. None of them have any experience with a BCI device. A questionnaire is handed out at the end of the experiment with questions about how the participant felt during it, without giving more details.

The remaining four participants perform a copy-spelling task where the computer monitor highlights the target letter, which is the one that the subject needs to focus. Across the duration of the trial, the current target letter is informed at the bottom of the screen.

#### 3.6.2. Passive Modality

First for a passive modality, real P300 ERP templates obtained from a public dataset, are superimposed into the generated EEG stream of 4 subjects. A set of template ERPs is acquired from the Subject Number 8 of the public dataset 008-2014 [69] published on the BNCI-Horizon website [70] by IRCCS Fondazione Santa Lucia. The experimental protocol implemented to produce this dataset is the same as the one described in Section 3.6.1. On the other hand, the EEG traces where these templates are superimposed, are experimentally obtained by subjects which are observing the flashing of the stimulus matrix during a P300 Speller procedure but they do not engage in focusing on any letter in particular. Everything is there, except the P300 ERP component. Hence, along the EEG stream, the markers information is used to localize the *True* segments where the P300 should be found, and those timing locations are used to superimpose the extracted ERP waveform. By implementing this pseudo-real approach, it is possible to effectively control null-signals and to adjust the shape of the evoked potential.

**Figure 2:**
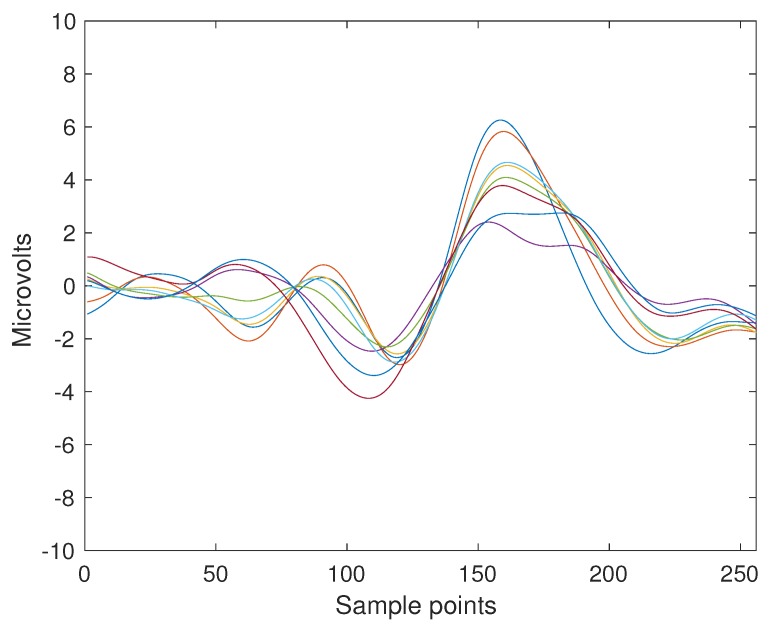
ERP Template obtained from the coherent point-to-point ensemble average from the signals of Subject Number Eight of the BNCI Horizon public dataset 008-2014. The template is 1 s long which is 256 sample points, and the eight channels are superimposed with different colors. The P3b component can be seen around the sample index 150 and 200.

A sample P300 ERP obtained from the trial number 2 of Subject 8 can be seen in Figure 2. These templates are selected due to their shapes more closely resembling the prototypical P300 waveform [71,72]. They are produced by extracting segments for this subject and by point-to-point coherently average them.

#### 3.6.3. Active Modality

Second, an active modality is also implemented, where a P300-Based BCI Speller experiment is performed on four subjects. For this scenario, the signal segments are modified to guarantee the inclusion of a P300 component. However, in this case the templates are extracted from the same subject. Hence, the EEG signal is preprocessed and labeled segments are extracted. Segments labeled *True* are coherently point-to-point averaged, and 70 templates are produced from the whole set of 35 trials.

Once templates are procured, a random *False* segment for the same subject is obtained. This is used as a baseline signal and is added to the template, conforming a new segment which has a superimposed P300 template. This procedure continues until the 700 segments marked as *True* are completed.

**Figure 3:**
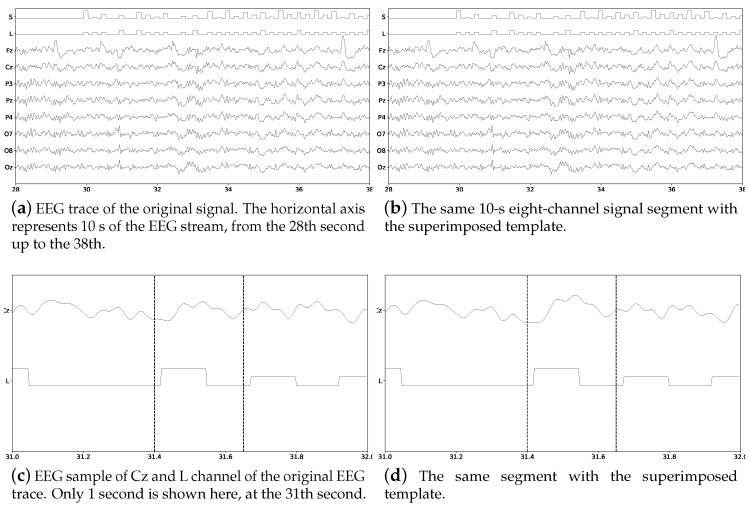
Eight-channel EEG signal for Subject Number 1 of the pseudo-real dataset without and with the superimposed ERP Template. The channel L, the mark which identifies where to superimpose the P300 ERP, is shown as well as the channel S which identifies the stimulus that was presented. On (**c**,**d**) the small variation that was introduced by the superimposition of the ERP can be seen enclosed by the vertical bars, where the slope of the bump on subfigure (**d**) is slightly bigger.

**Figure 4:**
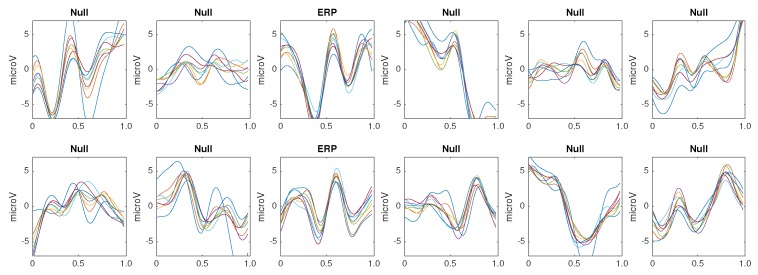
Point-to-point averaged signals. These are extracted from the first letter identification trial of the Subject 1 of the pseudo-real dataset. The ERP is superimposed on classes 3 and 9. Class 3 is obtained while averaging the segments where the row of the speller matrix is intensified whereas class 9 is calculated from the intensification of the corresponding column.

Figure 3 shows a 5 s sample of the EEG trace obtained with the MNE library [73]. Channel *S* represents the twelve different stimulus markers (columns or rows) while channel *L* represent the label (*True* vs *False*). Labels are represented by square signals. *False* segments are marked with single amplitude square signals while *True* segments are identified by double-amplitude square signals. Subfigure (a) shows the signals before the ERP template is superimposed while subfigure (b) shows the same signals with the superimposed ERP template. At first-sight, differences are really hard to spot visually. Subfigures (c) and (d) show only one second of channels Cz and L from the same segment. The superimposed ERP can be devised enclosed by the vertical bars, around 31.5 s, where in (d) the peak is slightly bigger. Figure 4 shows the obtained ensemble average ERPs as result of superimposing the template signal into the EEG stream, time-locked to the stimulus onset. These 12 point-to-point averaged segments correspond to the first trial of the EEG stream.

#### 3.6.4. Experiments

The experiments are as follows:

- Experiment 1—Letter Identification Performance: the letter identification performance of each one of these methods on the artificially generated pseudo-real dataset. The pool of 70 P300 ERP waveforms, either obtained from the same subject in the passive-modality or from each subject in the active-modality are used to compose the artificial P300 wave in the pseudo-real dataset. Templates are randomly selected.
- Experiment 2—Latency Noise: Instead of superimposing the P300 ERPs over the EEG trace at the exact locations where stimulus onsets are situated, an artificial latency lag is added. The lagging value is picked from a uniform distribution U(0, 0.4) [s] ranging from 0 to 0.4 of the 1 s segment size [74].
- Experiment 3—Component Amplitude Noise: the amplitude of the main P3b component of the ERP template is randomly altered. This component is defined to be located from the stimulus onset between 148 ms up to 996 ms which is around 840 ms long. This waveform element, multiplied by a gain factor, is subtracted from the original template. This gain factor between 0 and 1 is drawn from a uniform distribution U(0, 1). Additionally this subtracted waveform is multiplied by a Gaussian window with a support of the same length [75]. This avoids adding any discontinuity into the artificial generated signal.

All these experiments are executed using cross validation procedure dividing the letter to spell in two sets, preserving the structure of the letter identification trials. Spelling letters are scrambled while the order and group of each intensification sequence is preserved.

Finally the performance at letter identifications for these same methods is evaluated by running an offline BCI Simulation on the Dataset IIb of the BCI Competition II (2003) [76]. The protocol of this dataset is very similar to what was used to obtain the pseudo-real dataset. The sampling frequency of this dataset is 240, the number of letters are 73 where the first 42 are used to create the template dictionary for all the methods and the remaining 31 are used to test the character recognition rate performance. Additionally, in this dataset the number of available intensification number sequences is 15. The classification method Support Vector Machine SVM with a linear kernel, is added for comparison as control using a feature *f* constructed by normalizing the signal on each channel [77]. This method has been proved efficient in decoding P300 in several BCI Competitions [78].

#### 3.6.5. Classification

**Figure 5:**
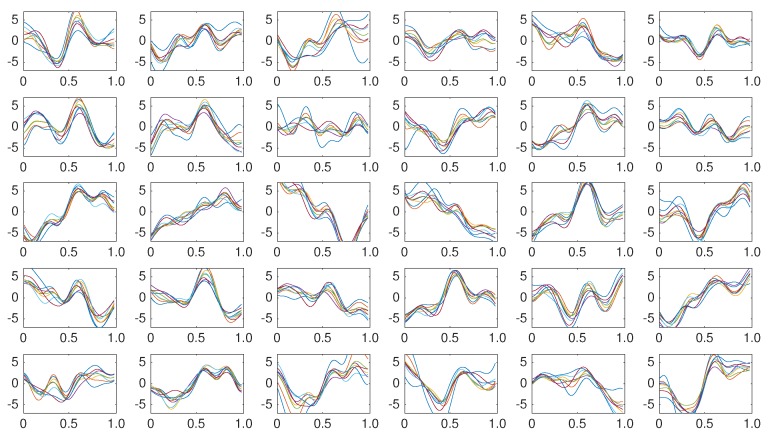
Coherently averaged signals segments of 1-second length containing the superimposed ERP. Vertical axis unit is μV. Each one is extracted from the EEG signal of the Subject 1 of the pseudo-real dataset. These averaged signals correspond to the 15 first trials (2 averaged signals from each trial, one belonging to the column flashing and the other to row flashing). These are the templates used to build a dictionary per channel per subject and are used by the classification algorithm described in Section 3.6.5.

The same classification algorithm based on k-nearest neighbors is used for all the methods [79]. The experimental protocol used to generated the pseudo-real dataset used in the experiments 1 to 3 is composed of 35 trials to spell 7 words of 5 letters each. Each trial is composed of 10 intensification sequences of the 6 columns and 6 rows of the Speller Matrix. Fifteen trials are used to build the dictionary of templates, extracting the averaged EEG segments for the row and column that already contain the P300 ERP, hence shielding 30 different templates per channel. Figure 5 shows the set of templates while using the first 15 trials of the dataset.

Described algorithms produce a feature *f* for each averaged EEG segment. The aim of the classification procedure is to identify for the remaining 20 trials which of the 6 features *f* that were obtained for row intensification, labeled by {1, …, 6}, and which of the 6 features for column intensification, named {7, …, 12} are the ones that elicited the P300 response on the averaged EEG segment. The row number of the matrix can be obtained by doing

(27) $$\hat{row}=\arg\min_{u\in \{1,\ldots ,6\}}\sum_{i=1}^{k}{∥f_{u}-q_{i}∥}^{2}$$

with $q_{i}$ being the set of k-nearest neighbors of the feature $f_{u}$ with *u* varying from 1 to 6. The parameter *k* represents the number of neighbors chosen from the dictionary of templates. The column can be obtained in the same way,

(28) $$\hat{col}=\arg\min_{u\in \{7,\ldots ,12\}}\sum_{i=1}^{k}{∥f_{u}-q_{i}∥}^{2}.$$

Thus, the letter identification performance can be obtained by measuring the accuracy channel-by-channel at identifying the correct letter on the matrix, coordinated by $\hat{row}$ and $\hat{col}$.

## 4. Results

**Figure 6:**
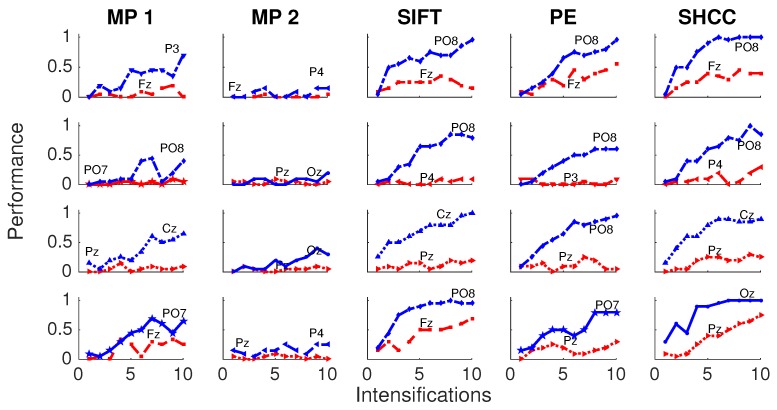
Passive Modality—Experiment 1: Speller performance curves obtained for each method for the four subjects that performed the passive modality protocol. Y-axis shows performance accuracy while X-axis shows the number of intensification sequences used to calculate the point-to-point signal average. The two curves show the performance for the best and worst performing channel.

**Figure 7:**
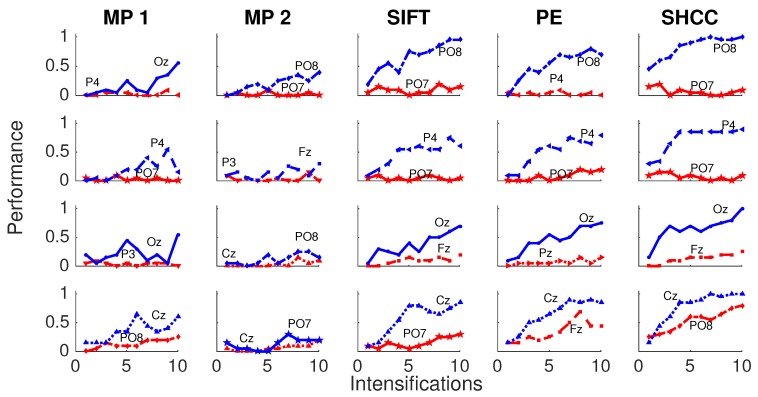
Active Modality—Experiment 1:Speller performance curves obtained for each method for the four subjects that performed the active modality protocol. Y-axis shows performance accuracy while X-axis shows the number of intensification sequences used to calculate the point-to-point signal average. The two curves show the performance for the best and worst performing channel.

Results for the first experiment are shown in Figure 6 and Figure 7. The performance while identifying each letter of the standard P300 Speller Matrix, and the channels where the best and worst performance are attained, are shown. Each one represents the percentage of letters that is actually predicted by the algorithms using a cross-validation procedure. As previously described the data is continuously divided in two sets, where the first 15 letters are used to derive the dictionary of templates while the remaining 20 letters are used to measure the letter identification performance. This is repeated one hundred times, and performances averaged. Figure 6 shows the results for the passive modality while Figure 7 shows the results for the active modality.

**Figure 8:**
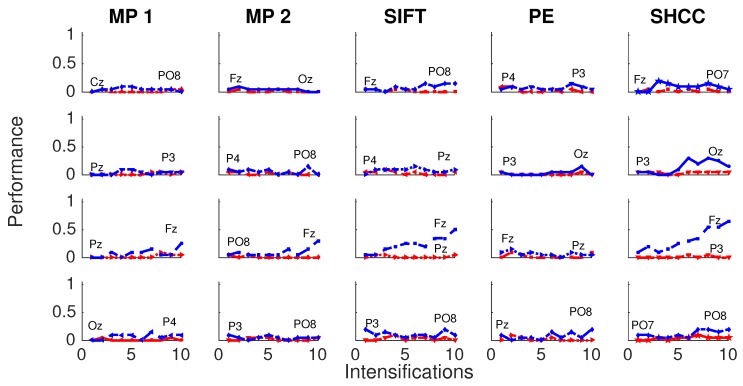
Passive Modality—Experiment 2: Performance curves for four subjects for the five algorithms when a random latency is included when superimposing the P300 signal template.

**Figure 9:**
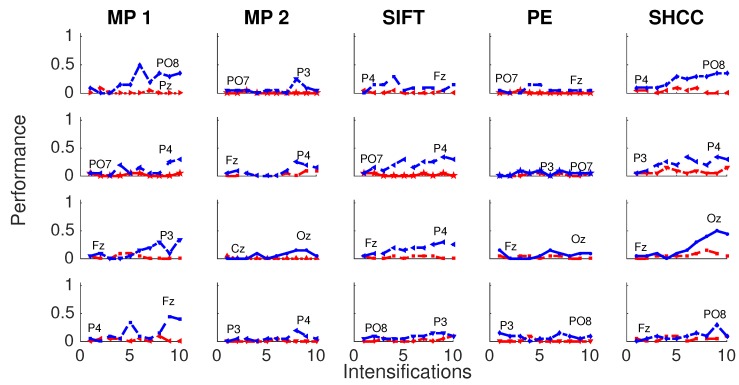
Active Modality—Experiment 2: Performance curves for the four subjects for the five algorithms. A random latency is included while superimposing the P300 signal template.

Figure 8 and Figure 9 shows the performance curves for five algorithms for the second experiment, where a noisy latency lag is included. Best and worst channels are also shown.

**Figure 10:**
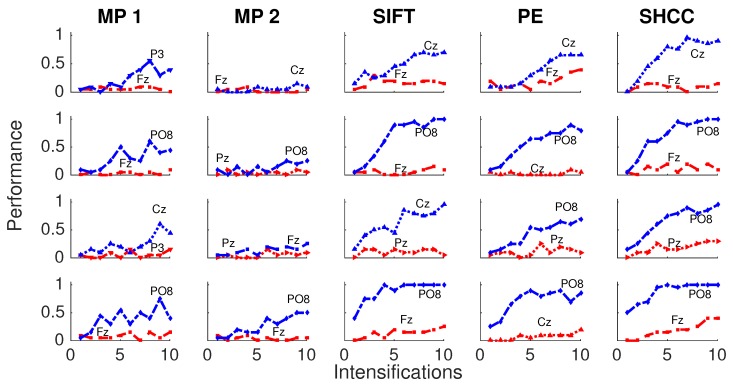
Passive Modality—Experiment 3:Performance curves for four subjects for the five algorithms when the amplitude of the P3b component of the template is randomly attenuated.

**Figure 11:**
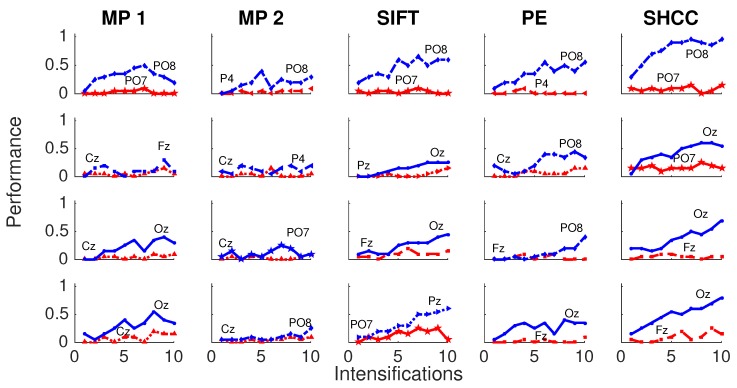
Active Modality—Experiment 3:Performance curves for four subjects for the five algorithms when the amplitude of the P3b component of the template is randomly attenuated.

Finally, Figure 10 and Figure 11 represents the performance values obtained for the Experiment 3, when the amplitude of the P3b component of the template is randomly attenuated.

**Figure 12:**
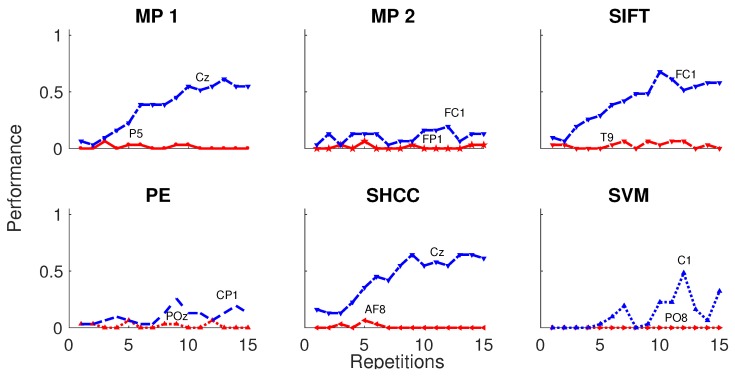
Speller performance obtained for the Dataset IIb of the BCI Competition II (2003) for each one of the algorithms. An offline BCI Simulation is performed using the first 42 trials as training and the remaining 31 as testing. The horizontal axis show the number of intensification sequences, from 0 to 15 for this dataset, while the vertical axis show the performance rate.

Furthermore, results obtained for the dataset BCI Competition 2003 IIb are shown in Figure 12 and in Table 2. For this experiment the number of available intensification sequences is 15.

## 5. Discussion

A significant reduction of performance was found when latency noise is added. The latency noise reduces the information contained in the averaged signal, mainly due to the invalidation of the SNR enhancement performed by the signal averaging procedure. This reduction alters the obtained shape of the waveform of the ERP and impacts on the performances regardless of the method. On the other hand, all the algorithms show some resistance to noise in peak amplitudes of the main component. This is shown by the similarities of obtained results between the Experiment 1 and 3.

Using a straightforward dictionary of templates for *MP-1* proved more beneficial in terms of performance than the approach of using a Hilbert base of Wavelets atoms on *MP-2*. Either applying latency noise or amplitude noise, the method based on the signal’s templates instead of using their coefficients achieve much better character identification rates.

Regarding results produced for the public and real dataset IIb of P300 ERP from the Berlin BCI Competition II (2003), the obtained character identification rate is above theoretical chance level, and for some algorithms close to the usable threshold of 70% [80,81]. When the character identification rate reaches this level of performance, the usage of word predicting algorithms allows to implement practical speller applications. Results for this competition have shown perfect classification with tailored algorithms [82]. This level is also similar to the performance obtained for the Experiment 3, which represents coincidentally the more realistic scenario for the pseudo-real dataset. It is important to remark that the algorithms presented here analyze the waveform structure of a single-channel signal [65,83].

## 6. Conclusions

The purpose of this work is threefold, (1) raise awareness about the utility of using automatic waveform-based methods to study EEG signals, (2) to provide an overview of the state-of-the-art of those methods, and (3) to compare those methods and verify if it is possible to obtain acceptable classification performances based exclusively on the signal’s waveform.

The higher performance results are obtained for the methods *SHCC* and *SIFT* either on the pseudo-real dataset and on the BCI Competition. We verified that it is possible to obtain discriminating information from the underlying signal based exclusively on an automated method of processing the waveforms. This brings the possibility to use these techniques to implement intelligible [84] automatic detection procedures, i.e., systems that are able to emphasize clearly and noticeable what are the factors that caused the system action, decision or classification. This is due to the fact that they are based on metrics which can be visually verified.

Further work should be conducted in terms of a multichannel meaningful extension of these waveform-based methods [83]. Moreover, the possibilities of finding overcomplete dictionaries for matching pursuit sparse representation based on obtained signal templates, could also be considered an area of future improvement.

We believe that the adoption of a *hybrid* methodology which can process the signal automatically, but at the same time, maintains an inherent intelligible property that can be mapped to existing procedures, and above all, can maintain the clinician trust on the system behavior is beneficial to Clinical Practice, Neuroscience and BCI research. Additionally, this may foster collaboration in a multidisciplinary environment and may ease the acceptance and translation of BCI technology [66]. The reason being, for caregivers and medical staff, particularly those with the expertise of the clinical EEG which is based on waveforms, they may feel a natural understanding of how the system is performing.

Another benefit of these methodologies is that they have a potential universal applicability. As they are only analyzing waveforms, they can be explored in other disciplines where the structure or shape of the waveform is of relevance. Analyzing signals by their waveforms is relative common in chemical analysis [85], seismic analysis in Geology [86], and quantitative financial analysis. Electrocardiogram EKG, on the other hand, has been extensively processed and studied analyzing the waveform structure [87].

## Figures and Tables

**Table 1:** EEG waveforms descriptions found in the surveyed literature.

Method	Phenomena	Reference
Positive Rounded Component	α-Waves, Epilepsy	[5,28]
Rising and Falling Phase	Epilepsy	[14,28]
Terminal plateau	Epilepsy	[14]
Ripples and Wiggles	Epilepsy, ERP	[14,26,29,30]
Sinusoidal Shape	Epilepsy	[19,28,29,30,31]
Sawtooth	Motor Imagery, Sleep	[22,26,28]
Sharpness or Spike-like	Epilepsy	[8,14,26,32]
Rectangular	Epilepsy	[14,19]
Line length	Anomaly Detection	[33]
Root Mean Square	Anomaly Detection	[33]
Wicket Shape	Epilepsy	[5,8,19,26,28,32]
Peak and Trough Sharpness Ratio	Epilepsy	[8,19,32,34]
Symmetry between rise and decay phase	Epilepsy	[8,19]
Slope Ratio	Sleep	[35]
Positive/Negative Peak Amplitude	ERP	[8,14,19,28,36,37]
Positive vs Negative Ratio	Sleep K-Complex	[26]
Base-to-Peak Amplitude	ERP	[19]
Peak-to-Peak Amplitude	ERP	[33,36]
Positive/Negative Peak Latency	ERP	[36]
Integrated Activity	ERP, Epilepsy, ICU	[25,33,38]
Cross-Correlation	ERP, Epilepsy, Sleep	[29,38]
Coupling
Cross Frequency, Phase-Amplitude, Phase-Phase	Sleep	[19]
Period Amplitude Analysis	ERP, Epilepsy	[25,29,38]

**Table 2:** Speller classification performance obtained for the dataset IIb of the BCI Competition II (2003) for each one of the algorithms using 15 repetitions of intensification sequences. The first 42 trials are used for training to build the template dictionary and the remaining 31 for testing. The channel where the best performance is attained, is also shown.

Method	Channel	Performance
MP 1	Cz	50%
MP 2	FC1	22%
SIFT	FC1	67%
PE	CP1	22%
SHCC	Cz	61%
SVM	C1	32%

## Abbreviations

The following abbreviations are used in this manuscript:

EEG	electroencephalography
BCI	Brain Computer Interfaces
BMI	Brain Machine Interfaces
BNCI	Brain-Neural Computer Interfaces
SNR	Signal to Noise Ratio
CNS	Central Nervous System
AC	Alternating Current
DC	Direct Current
ERP	Event-Related Potential
P300	Positive deflection at 300 ms
ITR	Information Transfer Rate
BTR	Bit Transfer Rate
SIFT	Scale Invariant Feature Transform
SHCC	Slope Horizontal Chain Code
PE	Permutation Entropy
MP	Matching Pursuit
ICU	Intensive Care Unit
EKG	Electrocardiogram
PAA	Period Amplitude Analysis
SVM	Support Vector Machine
